# Peptide Characterization and Functional Stability of a Partially Hydrolyzed Whey-Based Formula over Time

**DOI:** 10.3390/nu13093011

**Published:** 2021-08-28

**Authors:** Tristan Bourdeau, Michael Affolter, Lénaïck Dupuis, Alexandre Panchaud, Sabine Lahrichi, Loraine Merminod, Christine Martin-Paschoud, Rachel Adams, Sophie Nutten, Carine Blanchard

**Affiliations:** 1 Gastrointestinal Health Department, Nestlé Institute of Health Sciences, Nestlé Research, Société des Produits Nestlé S.A., Vers-chez-les-Blanc, 1000 Lausanne, Switzerland; Tristan.Bourdeau@rdls.nestle.com (T.B.); christine.martin.paschoud@gmail.com (C.M.-P.); 2 Analytical Sciences Department, Nestlé Institute of Food Safety & Analytical Sciences, Nestlé Research, Société des Produits Nestlé S.A., Vers-chez-les-Blanc, 1000 Lausanne, Switzerland; michael.affolter@rdls.nestle.com (M.A.); Alexandre.Panchaud@rd.nestle.com (A.P.); SabineLaila.Lahrichi@rdls.nestle.com (S.L.); loraine.merminod@gmail.com (L.M.); 3 Biometrics, Clinical Development Unit, Nestlé Research, Société des Produits Nestlé S.A., Vers-chez-les-Blanc, 1000 Lausanne, Switzerland; Lenaick.Dupuis@rdls.nestle.com; 4 Cultivate: Nutrition Content + Strategy, Decatur, TX 76234, USA; rachel.buchananadams@gmail.com; 5 Nestlé Health Science, 1000 Lausanne, Switzerland; Sophie.Nutten@nestle.com

**Keywords:** partially hydrolyzed whey-based infant formula, oral tolerance induction, hypoallergenic, peptide, atopic dermatitis risk reduction, allergy prevention

## Abstract

Human clinical trials have shown that a specific partially hydrolyzed 100% whey-based infant formula (pHF-W) reduces AD risk in the first yeast of life. Meta-analyses with a specific pHF-W (pHF-W1) confirm a protective effect while other meta-analyses pooling different pHF-W show conflicting results. Here we investigated the molecular composition and functional properties of the specific pHF-W1 as well as the stability of its manufacturing process over time. This specific pHF-W1 was compared with other pHF-Ws. We used size exclusion chromatography to characterize the peptide molecular weight (MW), a rat basophil degranulation assay to assess the relative level of beta-lactoglobulin (BLG) allergenicity and a preclinical model of oral tolerance induction to test prevention of allergic sensitization. To analyze the exact peptide sequences before and after an HLA binding assay, a mass cytometry approach was used. Peptide size allergenicity and oral tolerance induction were conserved across pHF-W1 batches of production and time. The median MW of the 37 samples of pHF-W1 tested was 800 ± 400 Da. Further oral tolerance induction was observed using 10 different batches of the pHF-W1 with a mean reduction of BLG-specific IgE levels of 0.76 log (95% CI = −0.95; −0.57). When comparing pHF-W1 with three other formulas (pHF-W2 3 and 4), peptide size was not necessarily associated with allergenicity reduction in vitro nor oral tolerance induction in vivo as measured by specific IgE level (*p* < 0.05 for pHF-W1 and 2 and *p* = 0.271 and *p* = 0.189 for pHF-W3 and 4 respectively). Peptide composition showed a limited overlap between the formulas tested ranging from 11.7% to 24.2%. Furthermore nine regions in the BLG sequence were identified as binding HLA-DR. In conclusion, not all pHF-Ws tested have the same peptide size distribution decreased allergenicity and ability to induce oral tolerance. Specific peptides are released during the different processes used by different infant formula producers.

## 1. Introduction

Allergies represent a public health concern in numerous countries worldwide [1,2]. Food allergies, atopic dermatitis (AD), and respiratory allergies affect an estimated 20% of the global population [1,2]. While the etiology of allergic manifestations is still unclear, clinical signs of atopic dermatitis (AD) often predate the development of asthma and allergic rhinitis later in life, also referred to as the atopic march [3,4]. Around one-third of patients with AD goes on to develop asthma and two-thirds develop allergic rhinitis. Symptom severity in AD positively correlates with decreased health-related quality of life (HRQoL) and increased healthcare costs [5].

While there are no obvious solutions for the prevention of allergy, international scientific societies have recommended strategies for allergy risk reduction. Exclusive breastfeeding is recommended for at least three to four months [6] or four to six months of life [7,8,9]. Maternal avoidance of highly allergenic foods is not recommended during pregnancy or lactation [6,7,8,9]. Complementary foods can be introduced between four and six months of age and some societies note that there is no evidence supporting a need to delay the introduction of highly allergenic complementary foods (such as peanut-containing foods) beyond four to six months, once a few typical complementary foods are tolerated [6,7,8,9]. For infants with a family history of allergy and who cannot be exclusively breastfed for the first four to six months, a 100% whey protein-based partially hydrolyzed formula (pHF-W) has been recommended [7,8], as several meta-analyses show a significant decrease in the risk of developing atopic dermatitis in at-risk infants [10,11] and infants from the general population [12] during the first year of life. Yet there are conflicting data in the literature and more well-designed clinical studies are necessary.

The methods and degree of protein hydrolysis differ between infant formulas and may explain why not all meta-analyses support the use of partially or extensively hydrolyzed formulas for primary allergy prevention [13,14]. Using a rat model of allergic sensitization, feeding with pHF-W before sensitization can prevent the rise in immunoglobulin E (IgE), demonstrating the preventive effect of a specific pHF-W (pHF-W1) on allergy development [15] in vivo. However, giving an extensively hydrolyzed whey-based formula (eHF-W) did not prevent allergic sensitization [15] in preclinical experiments. This raises the question of whether the method of hydrolysis may be as important as the degree of hydrolysis in allergy prevention, as evidenced by the lack of benefit from the eHF-W compared to the pHF-W1 in the German Infant Nutrition Intervention (GINI) studies [16,17].

Ideally, infant formulas would promote oral tolerance while minimizing sensitization. Decades of investigation have sought to understand how to induce oral tolerance in at-risk formula-fed newborns. In rat models, feeding pHF has been demonstrated to promote induction of oral tolerance, whereas some eHF failed to do so [15,18]. These early, preclinical results indicated that smaller peptides, derived from extensive hydrolysis, were not tolerogenic.

Tolerogenic peptides seem to require the right balance in size, sequence, structure, and dose [15]. European Commission Directive 2006/141/EC allowed certain infant formulas to claim a reduced risk of allergy to milk proteins [19] on the condition that “The infant formulae administered orally must not induce sensitization, in animals, to the intact proteins from which the infant formulae are manufactured.” An important consideration is how changes in manufacturing processes used to hydrolyze protein over time may have impacted the tolerogenic and allergenic properties of these formulas. This study explores the links between peptide characterization and oral tolerance induction to β-Lactoglobulin (BLG). BLG is known to be one of the major cow’s milk allergens and assessment of anti-BLG IgE levels over time provides valuable information on the tolerogenic and allergenic properties of certain pHF and their stability over time.

## 2. Materials and Methods

### 2.1. Formula Used

The description of samples pHF-W1, pHF-W2, pHF-W3, and pHF-W4, the intact protein formula (IF) and the extensively hydrolyzed whey formula (eHF-W used in this study (including brand name, product name and the lot number) are described in Supplementary Table S1.

### 2.2. Peptide Profiling (Molecular Weight Distribution)

Size exclusion chromatography (SEC) with UV detection was used to evaluate the peptide molecular weight (MW) distribution in pHF-W, according to the protocol described by Johns et al. [20], except for the protein and peptide standards used for MW calibration.

To obtain MW information for the peptide size distribution, the elution time axis was calibrated (cubic fitting) using the following 17 standard proteins, peptides and free amino acids: (1) serum albumin (bovine, MW ~66′354), (2) ovalbumin (chicken, MW ~42′750), (3) carbonic anhydrase (bovine, MW ~28′964), (4) beta-lactoglobulin (bovine, MW ~18′264), (5) alpha-lactalbumin (bovine, MW ~14′168), (6) ubiquitin (bovine, MW ~8′564), (7) insulin (bovine, MW ~5′733), (8) vasoactive intestinal peptide (VIP, mouse, MW ~3′325), (9) dynorphin A (porcine, MW ~2′147), (10) substance P (horse, MW ~1′347), (11) angiotensin II (human, MW ~1′046), (12) MRFA tetrapeptide (MW ~607), (13) IPP tripeptide (MW ~325), (14) VPP tripeptide (MW ~311), (15) IL dipeptide (MW ~245), (16) GGG tripeptide (MW ~189), (17) AA18 mix (mix of 18 amino acids, average MW ~110). SEC analysis is an established approach to estimate peptide size distribution of complex protein hydrolysates. The use of a diverse selection of proteins, peptides and amino acids allowed MW calibration of the elution time axis. In our determination, cubic fitting represented the best match between measured retention times and theoretical MW of the calibrants (data not shown). The fitting formula was then directly inserted into an Excel file and used for plotting the chromatograms, as well as the area under the curve (AUC) calculations. Any signal below 190 Da was excluded from the AUC integration as this represents the free amino acid mass range, which is not relevant for the peptide size distribution assessment.

### 2.3. Peptide-HLA-DR Binding Assay

Thp1 (ATCC, Manassas, VA, United States) human monocytic cells expressing HLA-DR were incubated at 2 *×* 10^7^ cells in RPMI-1640 medium with L-Glutamine (Sigma, Saint-Louis, MO, United States) supplemented with 10% heat-inactivated FCS (Amimed, Allschwil, Switzerland) and Penicillin/Streptomycin (Sigma, Saint-Louis, MO, United States) at a concentration of 1 × 10^6^ cells/mL in a T175cm2 culture flask with respectively 1 mg protein/mL or 100 mg protein/mL of pHF-W1 (Nestlé, Switzerland) for 24 h at 37 °C in presence of 5% CO_2_. Cells were spun down and harvested to perform immunoprecipitation of MHC-peptide complexes.

### 2.4. Protein Lysate and Immunoprecipitation

For each condition tested (with and control without pHF-W) 2 × 10^7^ cells were resuspended in ice-cold TBS 1X (Sigma-Aldrich Chemie GmbH, Buchs, Switzerland) and washed three times. The cell pellet was then resuspended in lysis buffer containing 1% CHAPS (Life Technologies Europe B.V., Zug, Switzerland) and proteinase inhibitors. After lysis, the supernatant was collected and mixed with antibody-PAS beads (rProtein A Sepharose Fast Flow, Sigma-Aldrich Chemie GmbH, Buchs, Switzerland) that were previously prepared by incubating 60 µL of PAS beads with 50 µg of MHC II monoclonal antibody specific for HLA-DR molecules (Clone L243, Santa Cruz Biotechnology, Heidelberg, Germany). Lysate and Ab-beads were incubated overnight at 4 °C with gentle rotation. Following affinity capture, beads were spun down and the supernatant discarded. Several washes were performed using respectively: 1 × 1 mL 1% CHAPS buffer, 1 × 1 mL 20 mM Tris/150 mM NaCl, 1 × 1 mL 20 mM Tris pH 8.0. After washing, 200 µL of 10% acetic acid solution was added to the beads, vortexed gently, and incubated for 15 min at 70 °C. Beads were spun down 5 min at 14,000× *g*. This step was repeated once. Supernatants were collected, pooled, and filtrated using a 10 kDa cut-off ULTRAFREE-MC centrifugal filter (Millipore Corporation, Marlborough, MA, USA). Both the retentate (containing mainly HLA-DR antibodies and HLA-DR molecules) and the filtrate (containing HLA-DR presented peptides) were kept at −20 °C for further LC-MS/MS analysis.

### 2.5. Mass Spectrometry Analysis

Before mass spectrometry analysis, HLA-DR peptides were desalted using either C18 (reversed-phase) or OASIS (mixed mode) desalting columns as described in the manufacturer’s manual (Waters Corp.). After desalting, peptides were analyzed by nano-LC-MSMS using an in-house Magic C18 column (150 × 0.075 mm) for peptide separation coupled to Orbitrap XL instrument (Thermo Fisher, Basel, Switzerland). Peptides were selected for fragmentation using data-dependent acquisition of the top five ions per each full scan. Dynamic exclusion was turned on with an exclusion duration of 30 s. Fragmentation was performed using CID at 35% collision energy. Peptides were finally identified by Sequest database search against a bovine milk database and manually validated based on spectrum quality and score.

### 2.6. BLG Allergenicity

The residual BLG allergenicity of the infant formulas was measured using an in vitro 3H-serotonin release assay. This method quantifies the release of serotonin from granulocytes after cross-linking of anti-BLG immunoglobulin E (IgE) antibodies, previously loaded on IgE-receptor, by residual BLG protein present in the product. In this assay, a rat basophil leukemia cell line, RBL-2H3 (ATCC, Manassas, VA, United States) was plated on 100 µL/well plate at 4 × 10^4^/well. After 2 h, cells were loaded with a rat hyperimmune serum (containing anti-BLG IgE) at one-half dilution in HBSS and with radioactive 5-hydroxytryptamine [3H] trifluoroacetate (Anawa, Kloten, Switzerland) 80 ci/mmol. 1 mci/37 mBq/mL. Cells were then stimulated by either different doses of BLG to generate a standard curve or different doses of the infant formula. Residual allergenicity was calculated at 50% of the maximum release and reported to 1 g BLG/g protein. This assay was modified from Fritsché et al. and Ballmer-Weber et al. [18,21]

### 2.7. Oral Tolerance Induction

An in vivo oral tolerance (OT) model was used to test different formulas for their potential to reduce sensitization to BLG and to induce immune tolerance. The partially hydrolyzed formulas were chosen as commercially available on the Swiss market at the time of the study (pHF-W1 batch L21080742C1, pHF-W2 batch HA1-21381878, pHF-W3 batch 21351391, and pHF-W4 batch C1277181000671). Seven to 10 weeks old Sprague Dawley rats (Charles River, Ecully, France) were fed ad libitum with drinking water (negative control) or pHF-W formula for 19 days. On day 5, rats were sensitized to BLG protein (Sigma Aldrich, L0130, Germany) via subcutaneous injections of 100 µg BLG emulsified in 1 mg Alum (Invivogen, San Diego, CA, United States) in saline solution to induce BLG specific IgE antibodies. Animals were euthanized under anesthesia on day 19 and blood was collected at the aorta at the hepatic level into 5 mL heparin-lithium tubes (Milian AG, Boswil, Switzerland). Plasma was stored at −20 °C until further analysis. Animal experiments were performed in compliance with Swiss Federal regulations under the study protocols VD2178.2, VD718.8, and VD2825.

### 2.8. Anti-BLG IgE in Plasma

To assess IgE levels in plasma, 96 well plates (Maxisorp, Nunc, Roskilde, Denmark) were coated overnight at 4 °C with BLG (Sigma-Aldrich Chemie GmbH, Buchs, Switzerland) at a concentration of 50µg/mL in carbonate coating buffer. Plasma sera were then serially diluted in PBS 1X (Sigma-Aldrich Chemie GmbH, Buchs, Switzerland) and added to the coated plate for one hour at room temperature. After washing four times with PBS 1X- 0.05% Tween 20 (VWR International GmbH, Dietikon, Switzerland), a primary mouse anti-rat IgE/MARE-1 antibody (MCA 193, Bio-rad Laboratories AG, Cressier, Switzerland) at a dilution of 1:1000 was incubated at room temperature for one hour before washing four more times with PBS 1X- 0.05% Tween 20. A secondary goat anti-mouse IgG-horseradish peroxidase (HRP) conjugated antibody (A 0412, Sigma Aldrich Chemie GmbH, Buchs, Switzerland) was added at a dilution of 1:1000 at room temperature for one hour. The revelation was performed after a final series of washes by adding 1-step Ultra TMB ELISA (LuBioScience GmbH, Lucerne, Switzerland) to the well and incubated in the dark at room temperature for 5 to 15 min. The reaction was stopped by adding ELISA stop solution (LuBioScience GmbH, Lucerne, Switzerland) and absorbance at 450 nm was immediately read with a Varioskan LUX (Fisher Scientific AG Reinach, Switzerland). Data were analyzed using GraphPad Prism 7.05 (GraphPad Softwares, San Diego, CA, USA).

### 2.9. Statistical Analysis

The exact Wilcoxon nonparametric statistical test was used to compare groups. Statistical analyses were performed using the software R 2.14.1 or higher (R Foundation for Statistical Computing, Vienna, Austria). Results with a *p* ≤ 0.05 were considered significant. Data are expressed in log scale and associated 95% CI. Figures are representative of one confirmatory experiment. The meta-analysis was performed on 10 preclinical trials conducted from 2010 to 2015. Several batches of pHF-W1 were tested in those 10 trials. Inclusion criteria mandated that at least one pHF-W had to be used in a treatment arm and that all trials used the same validated model of oral tolerance induction, which included a significant reduction in BLG-specific IgE levels between sensitized animals given the positive control formula (pHF-W) versus the sensitized animals given H_2_O (i.e., negative control). A random-effect method was used to pool the data to account for the moderate variability between trials (I^2^ = 30%). A single comparison based on a combined effect [22] was considered in the case where two batches of pHF-W1 were tested within the same trial (trial H) to overcome unit-of-analysis errors.

## 3. Results

### 3.1. Peptide Size Distribution Is Conserved in the pHF-W over Time

**Figure 1:**
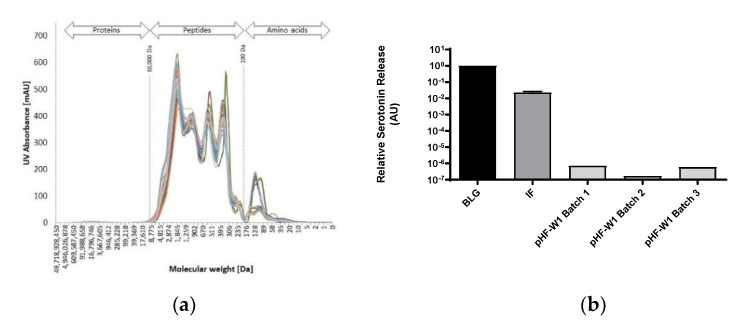

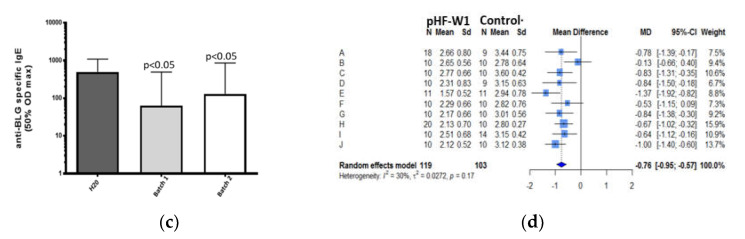
Peptide profile by size exclusion chromatography (SEC) of different batches of pHF-W1 (**a**); serotonin release test using primed RBL cells with different batches of pHF-W1 compared to BLG native protein and standard (non-hydrolyzed) cow’s milk IF (**b**); Quantification (ELISA) of anti-BLG IgE antibodies in BLG sensitized rats orally fed with different batches of pHF-W1 for 19 days compared to the negative control (H_2_O) (**c**); and meta-analysis of 10 preclinical trials investigating the reduction of BLG sensitization using 12 different batches of the same pHF-W1 result are mean differences (MD). Both standard deviation (Sd) and confidence interval are shown. All experiment were performed with *n* ≥ 8 (**d**).

To assess whether the peptide size distribution of pHF-W1 was conserved over time and that clinical trials performed earlier still substantiate the current product, the molecular weight distribution of 27 batches of pHF-W1 produced over 10 years (between 2001 and 2014, see Supplementary Table S1) were analyzed and are shown in Figure 1a. When determining the average peptide size distribution for each pHF-W sample, the AUC was integrated (190–10,000 Da). The median MW (the value that splits the total AUC at 50%) of these 27 samples was 800 ± 400 Da. Of note, depending on local or regulatory requirements, more or less free amino acids were added, explaining variation observed for the peak corresponding to the “AA” range (<190 Da). These data suggest a conserved overlap in peptide size profile over time.

### 3.2. BLG Allergenicity of Different pHF-W1 Batches Is Consistently Decreased Compared to Intact BLG

To assess the consistency of residual BLG allergenicity over time, we tested different pHF-W1 commercial batches for in vitro allergenicity (Figure 1b). The three samples have been compared to intact BLG protein (normalized to 1) and to an infant formula (IF) composed of intact proteins (60–70% whey and 30–40% casein). The results show a decrease of more than 3 logs (1000 fold) of the relative serotonin release for the three different batches of pHF-W1 suggesting a consistent decrease of in vitro BLG allergenicity.

### 3.3. Reproducibility of Anti-BLG IgE Decrease Using Different Batches of pHF-W1 in an In Vivo Oral Tolerance Model

An oral tolerance induction model was used to assess whether pHF-W1 could reduce the level of sensitization to BLG (Figure 1c,d). When two batches of pHF-W1, labeled as Batch 1 and Batch 2, were administered orally to rats, a profound reduction of 0.67 logs (95% Cl = −1.02; −0.32) of specific IgE level was observed (*p* < 0.05) as compared to the water group (negative control). When pHF-W1 from different production years were compared using meta-analysis of ten in vivo trials including twelve e batches of pHF-W1, we observed a significant global decrease of anti-BLG IgE levels (Figure 1d). The analysis included trials from the following years: A = Combined effect of two batches of pHF-W1 samples (presented in Figure 1c) 2011; B = 2011; C = 2011; D = 2012; E = 2012; F = 2013; G = 2013; H = Combined effect of two batches of pHF-W1 samples, 2014, I = 2015 and J = 2015. The plot shows individual results and the pooled result, obtained through a random-effects model. A significant reduction (0.76 logs (95% CI = −0.95; −0.57)) in BLG-specific IgE levels was observed after administration of pHF-W1. Altogether these results show a consistent and significant decrease of BLG-specific IgE pHF-W over a 5-year time frame.

### 3.4. Oral Tolerance Induction, Allergenicity and Peptides Characterization of pHF-W1 Compared with Other Formulas

**Figure 2:**
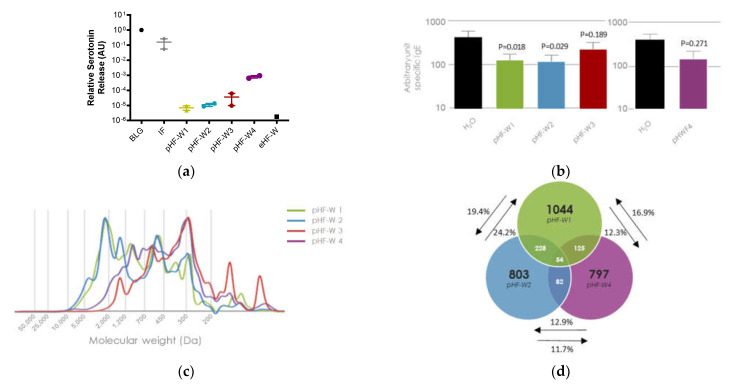
BLG residual allergenicity, assessed by a serotonin release test using primed RBL cells with different pHF-W compared to BLG native protein and standard (non-hydrolyzed) cow’s milk IF. Each point is the average of one batch measured in quadruplicate, the average of 2 independent product batches is shown, results with an extensively hydrolyzed formula (eHF-W) is shown as a reference (**a**); BLG-specific IgE antibodies in BLG sensitized rats orally fed with pHF-W1, 2, 3 and 4 for 19 days, two independent experiments are shown, *n* = 8 per group (**b**); Peptide profiles of pHF-W1, pHF-W2, pHF-W3, and pHF-W4 by size exclusion chromatography (SEC) (**c**); Venn diagram showing the number of specific peptides overlapping with pHF-W1, pHF-W2, and pHF-W4 peptide identified by mass spectrometry (**d**).

Over time, the consistency of the process for pHF-W1 has resulted in stable peptide size distribution, allergenicity decrease, and the ability to induce oral tolerance. We compared the pHF-W1 with three commercially available pHF-W from different manufacturers (Figure 2). Differences in the functionality of the different pHF-Ws were also observed based on in vitro assessment of the residual BLG allergenicity and on in vivo oral tolerance model. Assessment of the residual BLG allergenicity in pHF-W of different manufacturers demonstrates overall reduced allergenicity, but with various amplitudes (Figure 2a). While pHF-W1 and pHF-W2 demonstrated similar residual BLG residual allergenicity, higher allergenicity was observed in pHF-W4.

As previously described (Figure 1d), when pHF-W1 was administered orally to rats before allergen sensitization, we observed a significant reduction of plasma BLG-specific IgE (*p* < 0.05) as compared to the water group (negative control). A similar observation was found for pHF-W2 (*p* < 0.05), but not for pHF-W3 (*p* = 0.189) and pHF-W4 (*p* = 0.271) (Figure 2b).

Within the pHF-Ws, lower allergenicity was not associated with lower median size distribution nor reduced oral tolerance induction. The pHF-W3 and 4 which had a lower median peptide size (Table 1 and Figure 2c) had the highest residual allergenicity and did not induce oral tolerance induction (Figure 2a,b). The higher allergenicity is possibly resulting from residual long peptides or residual intact protein detected by ELISA or inhibition assays (data not shown), but not detected by SEC as shown in Figure 2c.

Differences were observed in the peptide profile characterizing the 4 pHF-W analyzed. Each pHF-W displays a specific peptide signature resulting from the different hydrolysis processes (Figure 2c). While different, pHF-W2 appears to be close in peptide size distribution to the pHF-W1 while pHF-W3 and pHF-W4 displayed a quite different peptide size distribution profile, resulting in a higher percentage of peptides with a molecular weight <1000 Da and a lower median weight (Table 1 and Figure 2c). pHF-W3 had a profile of an extensively hydrolyzed formula and was thus excluded for future analysis. When using mass spectrometry, the overlap between peptides (Figure 2d) of the three formulas with the higher median molecular weight, pHF-W1, pHF-W2, and pHF-W4, ranged from only 11.7% to 24.2% of the total peptides, suggesting that 75.8% to 88.3% of the peptides differ from one pHF-W to another.

These data suggest that biochemical characterization (peptide size profile and sequences) and in vitro (basophil degranulation) and in vivo (oral tolerance model) function may not always be associated.

### 3.5. Identification of Relevant Peptides in pHF-W1

**Figure 3:**
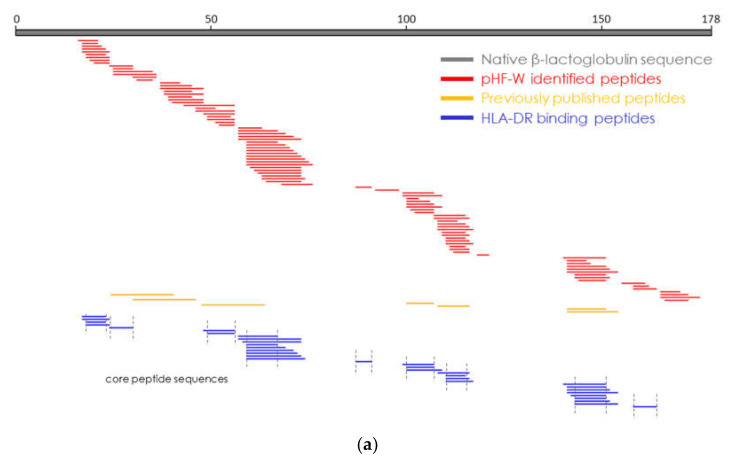

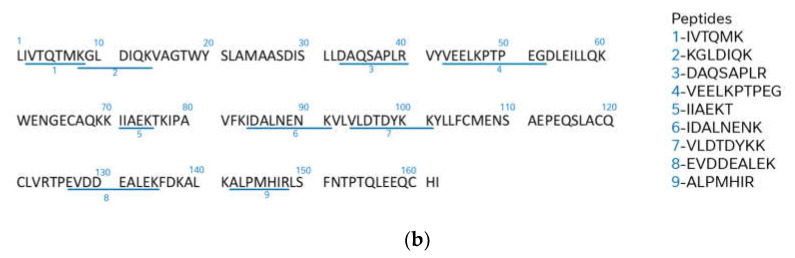
Results of the mass spectrometry analysis to assess bioactive peptides are shown. The top grey bar displays the sequence of the BLG, the main allergenic protein of the whey. All the peptides in the pHF-W1 and derived from BLG are represented by the red bars. Blue bars identify the peptides that were presented by the antigen-presenting cells. Previously published peptides position identified by Pecquet et al., 2015 [15], Meulenbroek et al., 2013 [23], and Gouw et al. 2018 [24], as being tolerogenic are displayed in orange (**a**). Exact sequences of the 9 core peptides identified are shown (**b**).

Oral tolerance induction to BLG was previously associated with specific peptides representing a limited coverage of the BLG amino-acid sequence. We aimed to identify peptides in pHF-W1, derived from BLG protein, that could potentially bind to MHC class 2 molecules. Using a human infant monocyte cell line (Thp1) followed by immunoprecipitation and mass spectrometry, we identified 33 peptides that could be detached from HLA-DR. These peptides were sub-grouped into nine sets of core peptides covering the majority of the BLG sequence (Figure 3). Interestingly, the HLA-DR binding peptides overlapped with those identified in pHF-W1, some of them having been previously identified as “tolerogenic” [15]. The data suggest that pHF-W1 contains specific peptides able to bind the MHC class 2 molecules and possibly educate the immune system. Here, we confirmed the presence of previously published “tolerogenic” peptides and identified new peptide core sequences (and locations) able to bind MHC class 2 molecules HLA-DR. These specific peptides, present in pHF-W1, may thus be key for the biochemical characterization and functionality of the pHF-W1.

## 4. Discussion

Whey-based partially hydrolyzed infant formulas are recommended for infants with a family history of allergy who cannot breastfeed [7,8]. The efficacy of pHF-W1 in reducing the risk of developing atopic dermatitis has been tested in numerous clinical trials [11,25]. Based on in vivo data, the mechanism by which this pHF-W1 works on allergy risk reduction appears to be two-fold: reducing allergenicity and inducing oral tolerance. Oral tolerance is an active immunologic acquisition that results in antigenic unresponsiveness. It is characterized by the suppression of local and systemic immune responses to innocuous food proteins [26]. When the body fails to induce oral tolerance, an allergic response may occur. Tolerogenic peptides require a balance between their size, sequence, structure, and dose [15]. There is a definite link between a peptide’s structure and function [15,20], yet this link is not yet fully understood.

BLG is known as the major antigen in cow’s milk. The use of BLG-derived peptides for oral tolerance has been evaluated in animal models [15,23,24,27]. BLG hydrolysate, peptides and, depending upon their size and structure, certain tolerogenic fractions can induce oral tolerance to native, intact BLG [15]. In this study, we assessed residual BLG in three batches of pHF-W1 and showed that BLG allergenicity was significantly reduced in all three batches of pHF-W1.

Antigenic and tolerogenic peptide sites appear to have distinct locations within milk protein sequences and play a key role in oral tolerance induction and sensitization potency [15,23,24]. The hydrolysis process is thus an important step in controlling the effect of the formula on allergy risk reduction. However, a delicate balance exists between the level of hydrolysis and tolerance. While it decreases allergenicity, hydrolysis can also decrease the presence or quantity of tolerogenic peptides needed for tolerance induction [15,18]. Too little hydrolysis could lead to sensitization, while too much may hydrolyze the tolerogenic inducers [15].

To investigate whether the size of the peptides might influence the tolerogenic effect, we compared three different pHF-Ws. We identified that the peptide size distribution of the formula could not predict allergenicity, nor its effect at inducing tolerance. Two of these formulas were very different (peptide profile) from the pHF-W1 used in the infant formula clinical trials. The two pHF-W with a lower MW (pHF-W3 and pHF-W4) did not show significant induction of oral tolerance. While this potential association between size and tolerogenic properties is supported by preclinical studies [20], human studies suggest a more complex mechanism of action. In the GINI study, prevention of eczema and asthma have been reported after early-life nutritional intervention with pHF-W1, but also with a casein-based extensively hydrolyzed formula [28]. This suggests that peptide size alone cannot explain functionality and formulas with similar peptide size distribution may not share more than 25% of peptide sequences and thus may not lead to similar biologic effects.

Beyond peptide size, the sequence and specific activity of peptides relative to immune modulation provide important information on the overall biological activity of infant formula [24]. Peptide sequences are influenced by protein source, enzymes employed, and the time and temperature of hydrolysis [24]. Whey contains numerous immunomodulatory peptides as part of their natural sequence that can be released during digestion or enzymatic hydrolysis [29,30]. These bioactive peptides can play a role in downstream immunological responses and cellular functions [29,31,32]. Thus, we analyzed the exact sequence of each peptide generated during the hydrolysis process to identify the bioactive peptides needed for the allergy prevention benefit (Figure 3). Very few overlapping peptides were identified among the pHF-W formulas tested, which suggested the infant formulas, while all partially hydrolyzed and whey-based, were quite different in terms of composition and function.

As mentioned previously, European Commission Directive 2006/141/EC allowed certain infant formulas to claim a reduced risk of allergy to milk proteins [19]. One of the conditions for the claim is “The infant formulae administered orally must not induce sensitization, in animals, to the intact proteins from which the infant formulae are manufactured.” A concern regarding this claim condition is whether changes in manufacturing processes over time would affect a formula’s ability to maintain this standard. This study sought to assess the stability of peptide profiles and the infant formula’s ability to prevent BLG sensitization in animals over time. While the representative rat model used in this study was subjected to variations over time (animal breeding or supplier, adjuvant supplier, housing facility), extremely good reproducibility of the capacity of partially hydrolyzed formula to induce oral tolerance was obtained. Results from this in vivo study and nine other in vivo trials were included in a meta-analysis of pHF-W1, which found that this pHF-W1 consistently and significantly reduces allergic sensitization compared to non-supplemented groups. These data, showing the stability of the process over time, support clinical trials results conducted over the past two decades on pHF-W1 demonstrating, with different batches of the pHF-W1, a risk reduction of atopic dermatitis when the formula is introduced within the first four months of life [17].

## 5. Conclusions

The hydrolysis process of proteins is a key determinant of their biological function associated with the generation of specific peptides. Peptide size alone, is too simplistic of an assessment of allergenic and tolerogenic potential. Peptide sequence, which is directly affected by the hydrolysis method, plays an important role in the ability of peptides to induce allergy reaction and/or immune tolerance. Data presented here and elsewhere demonstrate that not all pHF-W are the same regarding their tolerogenic potential. pHF-W differ in their peptide composition, and functionality. Thus, it cannot be assumed that all pHF-W reduce the risk of allergy. Tolerogenic potential and allergic sensitization risk must not be assigned to a type of formula, but rather only to formulas supported by relevant clinical studies.

The pHF-W1 is stable over time for peptide size distribution and function based on both in vitro and in vivo studies. Data presented in these studies provide further evidence that pHF-W1 contains HLA-binding peptides covering the BLG sequence, is tolerogenic, and is capable of reducing BLG-induced sensitization in an in vivo model. Further studies are warranted to better assess the biological functions of the hereby-identified peptides.

## 6. Patent

CB, SN, AP, MA, hold patents on partially hydrolyzed formula and bioactive peptides.

## Figures and Tables

**Table 1:** Peptide size distribution (in %) and median molecular weight (in Da).

	**>5000**	**5000–2500**	**2500–1000**	**<1000**	**Median MW [Da]**
pHF-W1	1.8	11.8	30.6	56.1	853
pHF-W2	3.2	12.9	29.9	54.4	804
pHF-W3	0.0	0.2	8.1	91.7	343
pHF-W4	0.8	1.7	16.8	80.9	465

## Data Availability

Data available on request.

## Supplementary Materials

The following are available online at https://www.mdpi.com/article/10.3390/nu13093011/s1, Table S1.
