# Supplementary material for: Peptide Characterization and Functional Stability of a Partially Hydrolyzed Whey-Based Formula over Time
Source: Nutrients. 2021 Aug 28;13(9):3011. doi: 10.3390/nu13093011 (PMC8465316; doi:10.3390/nu13093011)
Supplement: Supplementary file 1 [file nutrients-13-03011-s001.zip › nutrients-1321859-supplementary.pdf]

	Brand	Product name	Batch numbers	Protein source
**pHF-W2**	**Humana**	HA1	0022L-04.2014-21381878-02-07:15-008638	Hydrolyzed whey
	Humana	HA1	LL10G-09.10.10-21116993-01-20:20-000842	Hydrolyzed whey
	Humana	HA pre	MM25A-07.2011-21147998-01-09:09-003254	Hydrolyzed whey
	Humana	HA1	LL23I-03-2011-2128344-01-15:54-002366	Hydrolyzed whey
**pHF-W4**	**Milupa**	Aptamil HA 1	08.05.2014 C2 277239 001711	Hydrolyzed whey
	Milupa	Aptamil HA 1	24-05.2014 C1 277181 000671	Hydrolyzed whey
	Milupa	Aptamil HA 1	01.03.2011 L:3342232	Hydrolyzed whey
	Milupa	Aptamil HA pre	22.01.2001 L:2952007	Hydrolyzed whey
	Milupa	Aptamil HA pre	07.05.2009 L:3110816	Hydrolyzed whey
	Milupa	Aptamil HA 1	26.05.2009 L3301759	Hydrolyzed whey
	Milupa	Aptamil HA1	28-04-2011 C1 272572 000440	Hydrolyzed whey
**PHF-W3**	**Hipp**	HA plus Probiotik HA1	02.2011 L651110054 13:52 D	Hydrolyzed whey
	Hipp	HA1 Combiotik HA1	25.10.2013 21359978	Hydrolyzed whey
	Hipp	HA1 Combiotik HA1	25.10.2013 21359978	Hydrolyzed whey
	Hipp	HA plus Probiotiks preHA	10.2010 L651100020	Hydrolyzed whey
	Hipp	HA1	21351391	Hydrolyzed whey
**PHF-W1**	**Nestlé Nutrition**	BEBA HA2	2011 BEH 007, DEWBPKJAS	Hydrolyzed whey
	Nestlé Nutrition	BEBA HA1	2003 NAH 042, DEWBRRRPAZ	Hydrolyzed whey
	Nestlé Nutrition	GOOD START	2004 NWH001A-00S, 4265EWGPP1539 2212	Hydrolyzed whey
	Nestlé Nutrition	GOOD START	2004 NHW005-00, 4200EWG1P1433 0627	Hydrolyzed whey
	Nestlé Nutrition	NAN HA2	2002 DEWBUSKAU	Hydrolyzed whey
	Nestlé Nutrition	BEBA HA1	2005 NWHB 020-1, L-60760742CV	Hydrolyzed whey
	Nestlé Nutrition	NAN HA2 Milk Powder	2010 021107421B	Hydrolyzed whey
	Nestlé Nutrition	NAN HA2 Milk Powder	2010 0356074228	Hydrolyzed whey
	Nestlé Nutrition	NAN HA2 BL Gold	2009 9210074227	Hydrolyzed whey
	Nestlé Nutrition	NAN HA2 BL Gold	2010 00250742B5	Hydrolyzed whey
	Nestlé Nutrition	NAN HA2 BL Gold	2009 901807422X	Hydrolyzed whey
	Nestlé Nutrition	NAN HW Three BL Gold	2010 0038007421K	Hydrolyzed whey
	Nestlé Nutrition	NAN HA3 BL	2008 834507421M	Hydrolyzed whey
	Nestlé Nutrition	NAN HA3 BL	2009 918807421P	Hydrolyzed whey
	Nestlé Nutrition	NAN HA3 BL Gold	2011 10080742BK	Hydrolyzed whey
	Nestlé Nutrition	NAN HW BL Gold	2010 0203074211	Hydrolyzed whey
	Nestlé Nutrition	NAN HA1 B	2009 93030742AC	Hydrolyzed whey
	Nestlé Nutrition	BEBA HA START Pro B	2010 01290742BW	Hydrolyzed whey
	Nestlé Nutrition	BEBA HA START Pro B	2010 03410742AT	Hydrolyzed whey
	Nestlé Nutrition	NAN HA1 B	2009 913907421X	Hydrolyzed whey
	Nestlé Nutrition	BEBA HA1 B	2008 835607422C	Hydrolyzed whey
	Nestlé Nutrition	NAN-HA NWHB222	2010 L11867800292	Hydrolyzed whey
	Nestlé Nutrition	NAN-HA NWHB022-5A	040006664361 L025207423R	Hydrolyzed whey
	Nestlé Nutrition	NAN-HA NWHB200	118677 00085	Hydrolyzed whey
	Nestlé Nutrition	BEBA pro-HA NWHB022-5A	L013107424AW 040006254856 13833960	Hydrolyzed whey
	Nestlé Nutrition	BEBA HA1	L21080742C1	Hydrolyzed whey
**IF**				
	Nestlé Nutrition	Béba1 OPTIPRO	9097080622/NWSB007-100233940	Whey : casein
**eHF-W**				
	Nestlé Health Science	Althera	2012	Hydrolyzed whey
